# Supplementary material for: Hyphal Growth in Trichosporon asahii Is Accelerated by the Addition of Magnesium
Source: Microbiol Spectr. 2023 Apr 27;11(3):e04242-22. doi: 10.1128/spectrum.04242-22 (PMC10269644; doi:10.1128/spectrum.04242-22)
Supplement: Supplemental file 1 — Supplemental material. Download spectrum.04242-22-s0001.pdf, PDF file, 11.4 MB [file spectrum.04242-22-s0001.pdf]

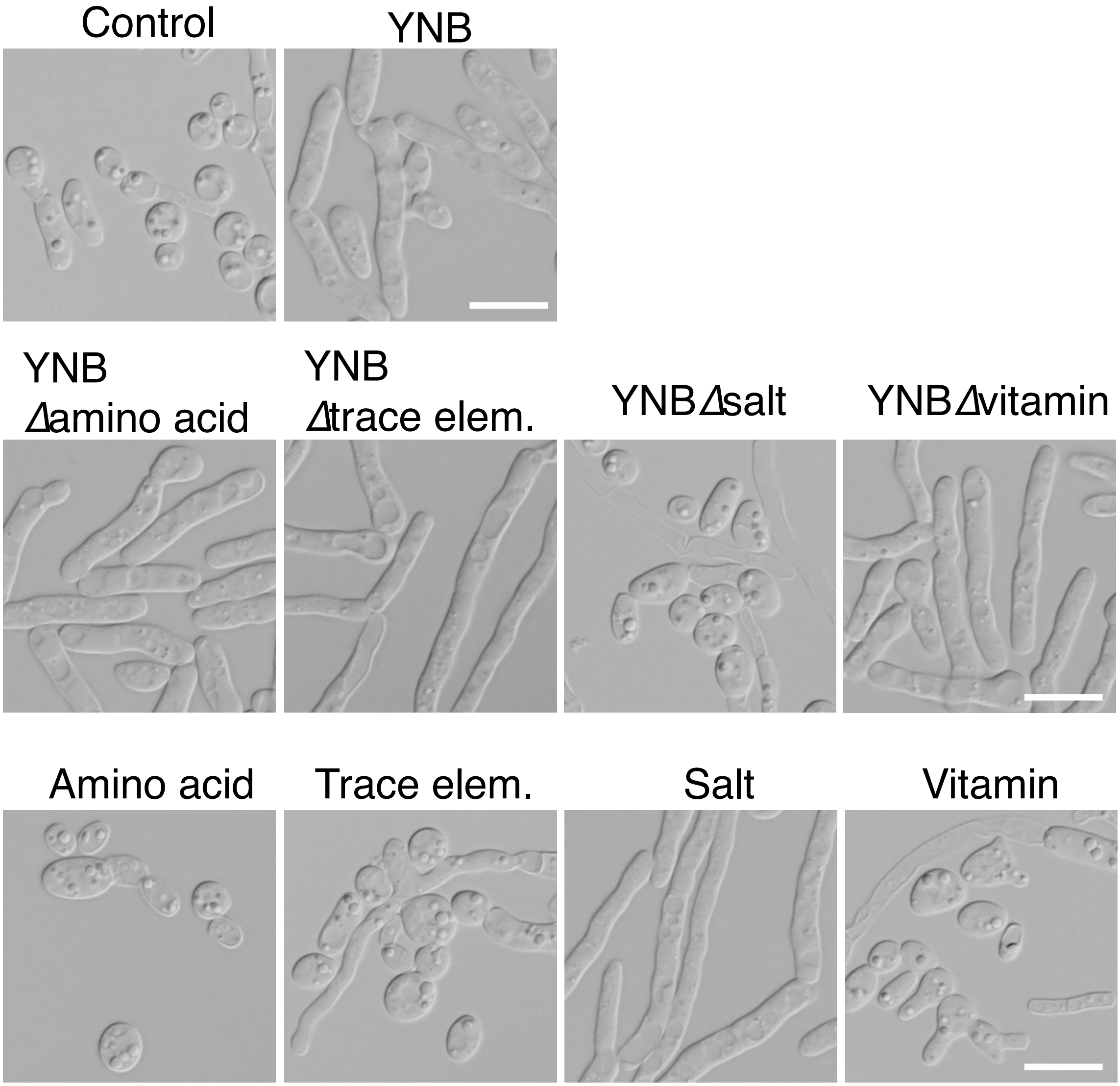

Cell phenotypes of JCM 2466 when supplemented with each ingredient of YNB.

Cell phenotypes of JCM 2466 cells after cultivation for 16 h at 25˚C in Sabouraud medium supplemented with each ingredient (amino acid, trace element, salt or vitamins) of YNB and each dropout of the ingredient were shown. Cell lengths were measured in Fig. 2A and Supplemental Table. The content of each group was described in Materials and Methods. Symbol Δ indicates dropout of the corresponding ingredient. Scale bar is 10 μm.

Supplemental Fig. 1

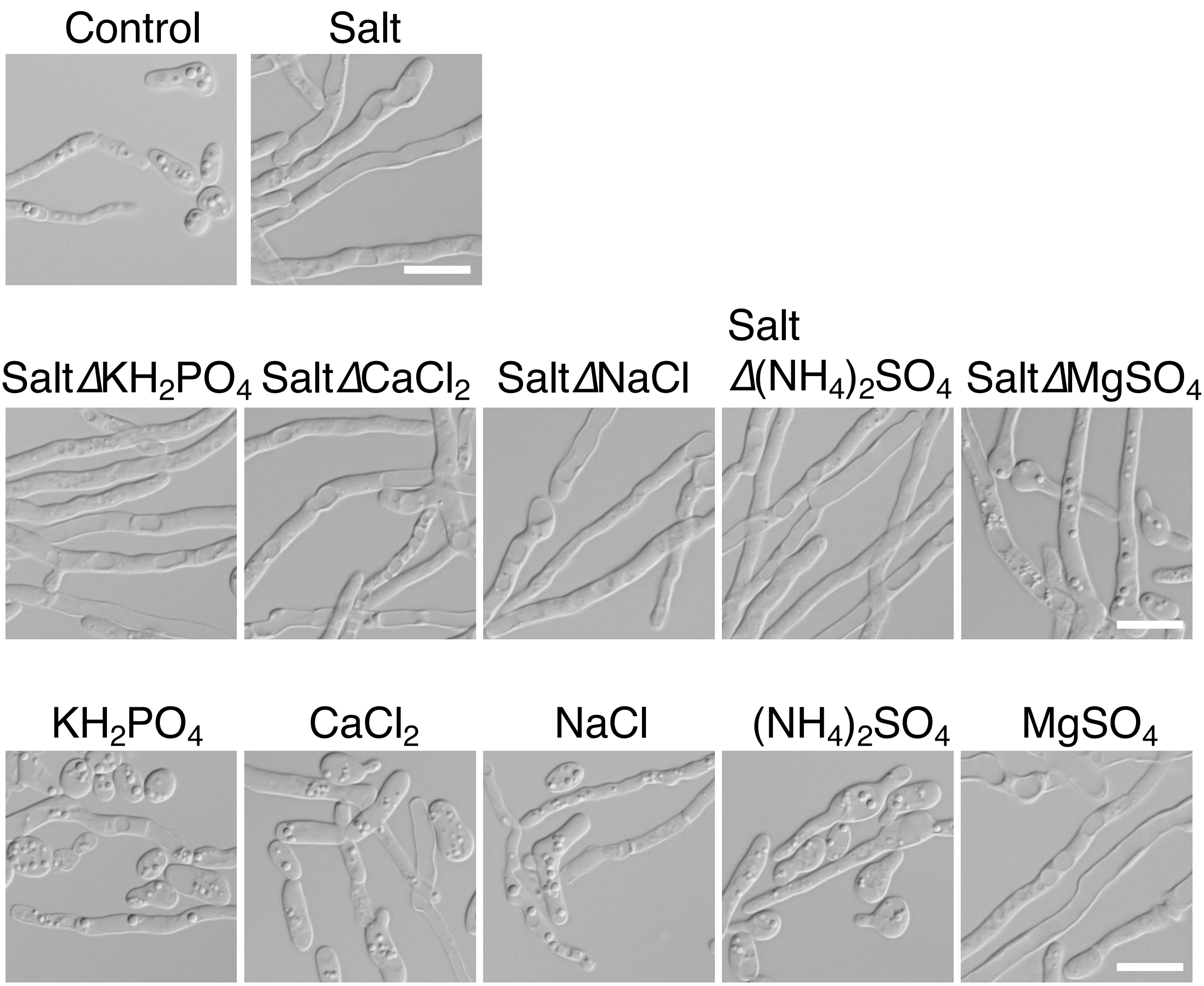

Cell phenotypes of JCM 2466 when supplemented with each ingredient of Salt.

Cell phenotypes of JCM 2466 cells after cultivation for 16 h at 25˚C in Sabouraud medium supplemented with each ingredient (KH_2PO_4, $CaCl_2$, NaCl, $(NH_4)_2SO_4$, or $MgSO_4$) of the salt group and each dropout of the ingredient were shown. Cell lengths were measured in Fig. 2B and Supplemental Table. Symbol Δ indicates dropout of the corresponding ingredient. Scale bar is 10 μm.

Supplemental Fig. 2

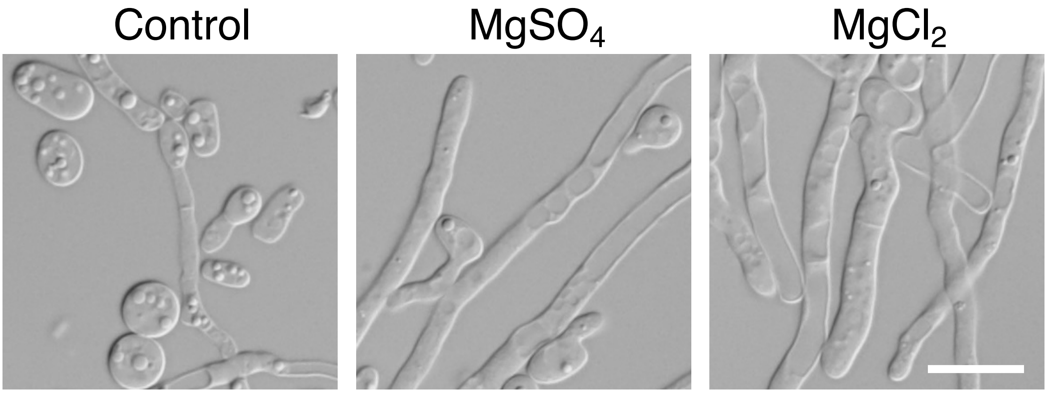

Hyphal growth was induced upon the addition of $MgCl_2$.
Cell phenotypes of JCM 2466 cultivated for 16 h at 25˚C in Sabouraud medium (control) and Sabouraud medium supplemented with 4.15mM $MgSO_4$ and 4.15mM $MgCl_2$ were shown. Cell lengths were measured in Fig. 2C. Scale bar is 10 μm.

Supplemental Fig. 3

A

Media	SYTOX	
	Positive	Negative
YPD	0.67 %	99.33 %
Sab	40.34 %	59.66 %
Sab+Mg	9.81 %	90.19 %

B

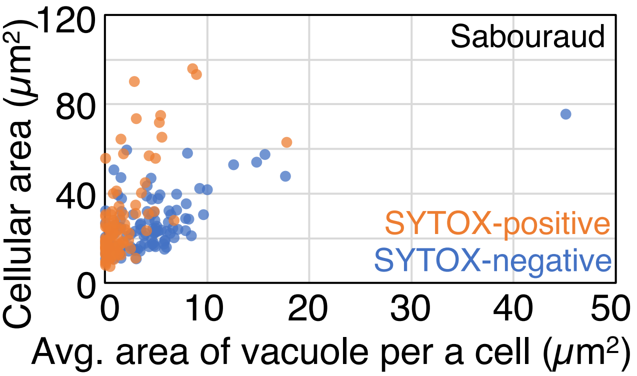

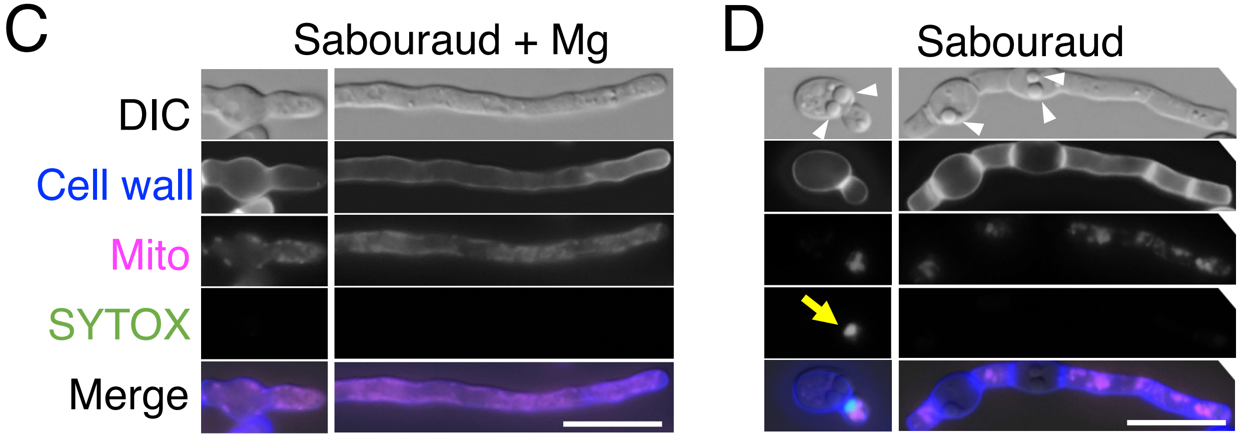

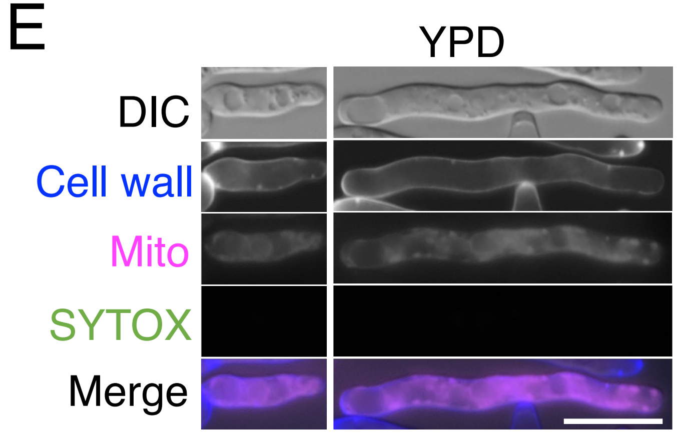

Dead cells were visualized by SYTOX Green staining.
(A) JCM 2466 cells were cultivated in YPD (N=300), Sabouraud (N=295), and Sabouraud+Mg (N=265) media for 16 h at 25˚C. The cells were stained by 5 μM SYTOX Green (Thermo Fisher Scientific, Waltham, MA, USA) to examine viabilities under a microscope. Frequencies of SYTOX-positive cells were shown.
(B) Area of vacuoles stained by FM4-64 was larger in SYTOX-negative cells than in SYTOX-positive cells ($p < 10^{-5}$) when cultivated in Sabouraud medium. (C, D, E) Mitochondria and cell walls were stained using MitoBright and Calcofluor White, respectively, at the same time as stained using SYTOX Green (an arrow), in each medium. Arrowheads indicated large lipid droplets. Scale bar is 10 μm. Sab, Sabouraud; Mito, mitochondria; DIC, differential interference contrast microscopy.

Supplemental Fig. 4

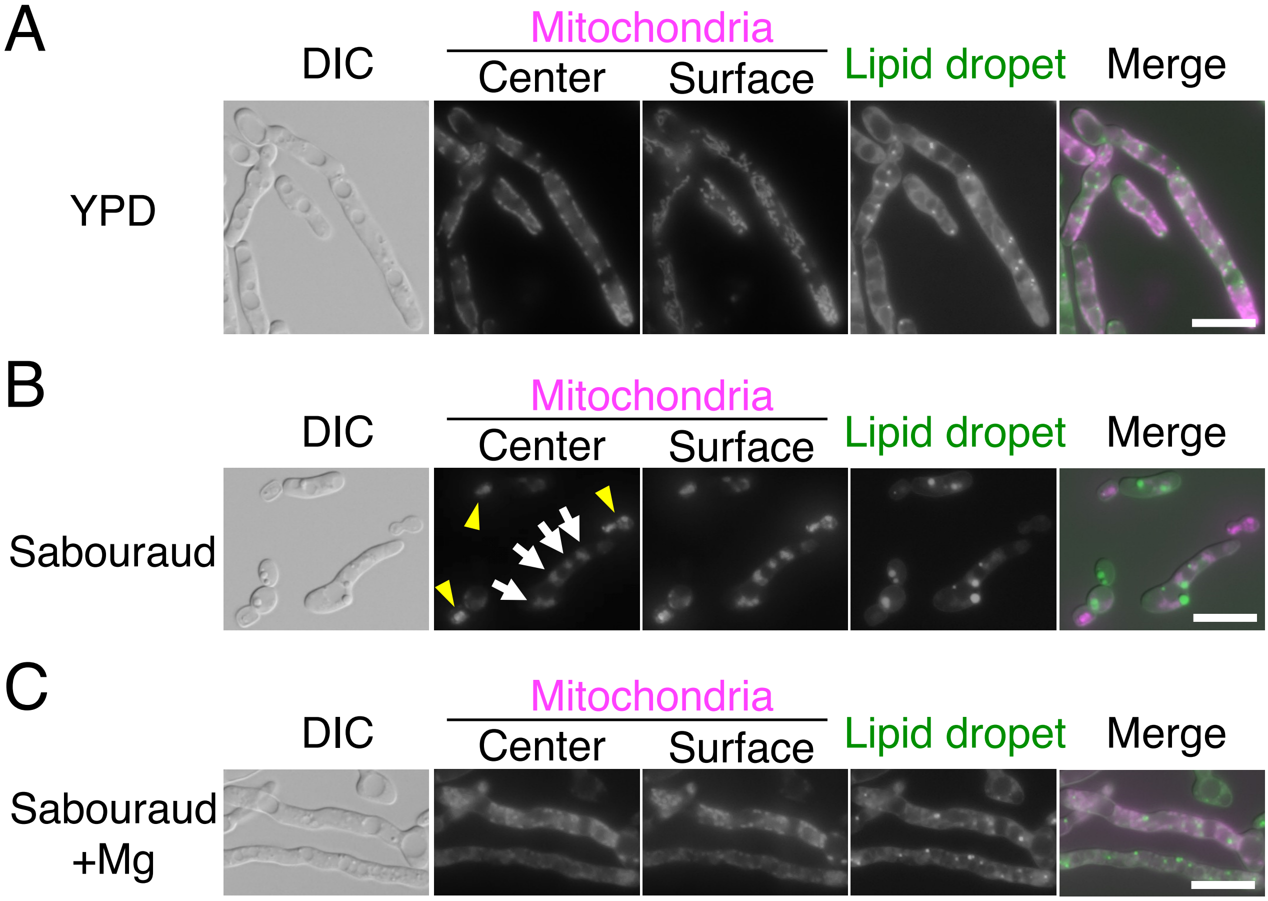

Ghost cells lacking the cytoplasm were appeared in Sabouraud medium in *T. asahii*.
Mitochondria and lipid droplets were stained with MitoBright and BODIPY, respectively, in JCM 2466 cells that were cultivated for 16 h at 25° C in (A) YPD, (B) Sabouraud, and (C) Sabouraud medium containing 4.15 mM $MgSO_4$. Center and surface view of mitochondria images indicate mitochondria distribution in the center and surface of a cell, respectively. Arrows indicate the fragmented mitochondria in a normal cell grown in Sabouraud medium. Arrowheads indicate ghost cells having the fragmented mitochondria. The scale bar is 10 μm. YPD, yeast extract-peptone-dextrose; DIC, differential interference contrast microscopy.

Supplemental Fig. 5

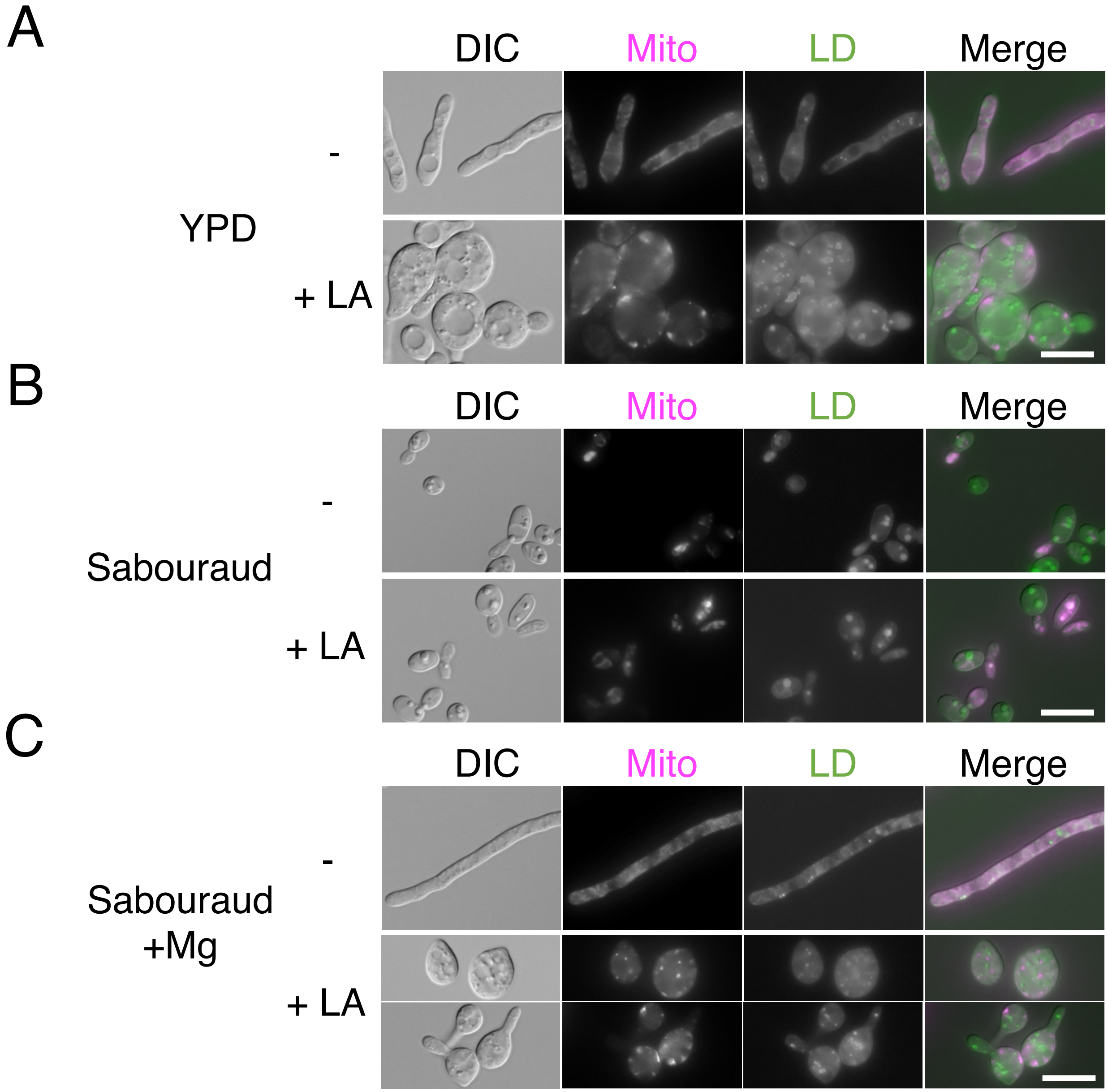

The mitochondrial distribution was disrupted when affected with an actin inhibitor.
JCM 2466 cells were treated with 10 μg/ml LA or DMSO (-) in (A) YPD medium, (B) Sabouraud medium, and (C) Sabouraud medium containing 4.15 mM $MgSO_4$ for 16 h at 25°C. Mitochondria and lipid droplets were stained using MitoBright and BODIPY, respectively. Scale bar is 10 μm. Mito, mitochondria; LD, lipid droplet; LA, latrunculin A; DIC, differential interference contrast microscopy.

Supplemental Fig. 6

Supplemental Table Summary of the measured values

	Item on the x-axis	Average (μm)	SD (μm)	Cell num.	Mann-Whitney U test (Significance *p*<0.05)	
					Against the control sample	Against each Δ sample
Fig. 2A	Control	8.41	8.96	N=262		ND
	All	20.61	13.41	N=252	*p*<0.00001 (z=-13.6)	ND
	YNBΔaminoacid	23.63	16.94	N=277	*p*<0.00001 (z=-15.9)	
	YNBΔmineral	29.17	24.56	N=278	*p*<0.00001 (z=-15.5)	
	YNBΔsalt	7.49	4.46	N=223	*p*=0.26272 (z=-1.12)	
	YNBΔvitamin	22.3	13.12	N=243	*p*<0.00001 (z=-15.2)	
	Aminoacid	8.66	9.92	N=308	*p*=0.71138 (z=0.37)	*p*<0.00001 (z=-16.5)
	Mineral	9.36	10.38	N=252	*p*=0.05 (z=-1.96)	*p*<0.00001 (z=14.5)
	Salt	31.8	30.67	N=229	*p*<0.00001 (z=-15.4)	*p*<0.00001 (z=15.3)
	Vitamin	9.12	9.68	N=263	*p*=0.86502 (z=0.17)	*p*<0.00001 (z=-14.4)

Cell length classification

	Item on the x-axis	0-10 μm	10-20 μm	20-30 μm	30-40 μm	40-50 μm	50-60 μm	60-70 μm	70-80 μm	80-90 μm	90-100 μm	>100 μm
Fig. 2A	Control	213	31	11	2	2	1	0	2	0	0	0
	All	56	85	62	31	7	6	3	1	1	0	0
	YNBΔaminoacid	34	98	84	32	13	11	1	0	1	1	2
	YNBΔmineral	55	76	48	34	15	19	17	5	3	1	5
	YNBΔsalt	187	29	6	1	0	0	0	0	0	0	0
	YNBΔvitamin	32	88	69	34	13	4	1	0	1	0	1
	Aminoacid	251	41	5	3	4	1	0	1	2	0	0
	Mineral	198	38	6	3	3	2	1	0	0	0	1
	Salt	32	55	53	40	16	6	7	9	5	0	6
	Vitamin	203	38	11	4	4	2	0	1	0	0	0

	Item on the x-axis	Average (μm)	SD (μm)	Cell num.	Mann-Whitney U test (Significance *p*<0.05)			
					Against the control sample	Against the SaltΔ$MgSO_4$ sample	Against the $MgSO_4$ sample	Against each Δ sample
Fig. 2B	Control	10.47	10.42	N=332		ND	ND	ND
	Salt	62.28	70.08	N=311	*p*<0.00001 (z=-16.8)	*p*<0.00001 (z=4.35)	ND	ND
	SaltΔKH_2PO_4	66.29	66.54	N=257	*p*<0.00001 (z=-17.1)	*p*<0.00001 (z=-5.97)	ND	
	SaltΔ$CaCl_2$	56.22	55.77	N=327	*p*<0.00001 (z=-17.8)	*p*<0.00001 (z=5.12)	ND	
	SaltΔNaCl	61.58	62.58	N=326	*p*<0.00001 (z=-18)	*p*<0.00001 (z=5.71)	ND	
	SaltΔ$(NH_4)_2SO_4$	84.05	73.26	N=243	*p*<0.00001 (z=-17.8)	*p*<0.00001 (z=-8.62)	ND	
	SaltΔ$MgSO_4$	43.77	52.89	N=282	*p*<0.00001 (z=-13.5)		ND	
	KH_2PO_4	9.86	10.21	N=255	*p*=0.40654 (z=-0.83)	ND	*p*<0.00001 (z=-17.1)	*p*<0.00001 (z=16.6)
	$CaCl_2$	21.21	23.02	N=320	*p*<0.00001 (z=-9.46)	ND	*p*<0.00001 (z=-12.7)	*p*<0.00001 (z=11.9)
	NaCl	11.18	10.72	N=297	*p*=0.0226 (z=-2.28)	ND	*p*<0.00001 (z=-16.9)	*p*<0.00001 (z=17.1)
	$(NH_4)_2SO_4$	15.83	15.99	N=302	*p*<0.00001 (z=-6.92)	ND	*p*<0.00001 (z=-14.8)	*p*<0.00001 (z=-15.6)
	$MgSO_4$	81.4	89.95	N=256	*p*<0.00001 (z=-17.6)	ND		*p*<0.00001 (z=-6.8)

Cell length classification

	Item on the x-axis	0-10 μm	10-20 μm	20-30 μm	30-40 μm	40-50 μm	50-60 μm	60-70 μm	70-80 μm	80-90 μm	90-100 μm	>100 μm
Fig. 2B	Control	234	56	19	13	6	3	0	1	0	0	0
	Salt	43	52	34	44	19	20	14	12	5	8	60
	SaltΔKH_2PO_4	19	43	32	31	18	17	12	12	8	8	57
	SaltΔ$CaCl_2$	29	56	56	36	20	18	20	18	18	5	51
	SaltΔNaCl	31	54	37	31	35	27	16	15	13	13	54
	SaltΔ$(NH_4)_2SO_4$	17	24	13	26	13	23	25	9	7	10	76
	SaltΔ$MgSO_4$	70	65	36	17	21	9	8	9	7	7	33
	KH_2PO_4	194	39	12	6	0	1	1	1	0	1	0
	$CaCl_2$	122	97	32	25	17	7	7	3	4	1	5
	NaCl	199	56	26	7	3	4	1	0	1	0	0
	$(NH_4)_2SO_4$	142	98	21	18	9	5	4	1	1	2	1
	$MgSO_4$	15	43	37	28	21	12	10	8	5	6	71

	Item on the x-axis	Average (μm)	SD (μm)	Cell num.	Mann-Whitney U test (Significance *p*<0.05)	
					Against the control sample	Against the $MgSO_4$ sample
Fig. 2C	Control	8.58	8.43	N=158		*p*<0.00001 (z=12.0)
	$MgSO_4$	44.59	45.99	N=199	*p*<0.00001 (z=12.01)	
	$MgCl_2$	46.16	52.85	N=196	*p*<0.00001 (z=11.51)	*p*=0.61006 (z=0.51)

Cell length classification

	Item on the x-axis	0-10 μm	10-20 μm	20-30 μm	30-40 μm	40-50 μm	50-60 μm	60-70 μm	70-80 μm	80-90 μm	90-100 μm	>100 μm
Fig. 2C	Control	128	21	3	4	1	0	1	0	0	0	0
	$MgSO_4$	41	35	28	18	21	10	6	4	7	2	27
	$MgCl_2$	45	41	20	19	11	11	12	3	6	2	26

	Item on the x-axis ($MgSO_4$ conc.)	Average (μm)	SD (μm)	Cell num.	Mann-Whitney U test (Significance *p*<0.05)
					Against the control sample
Fig. 2D	Control	8.34	9.15	N=231	
	x1 (4.15 mM)	38.41	31.58	N=232	*p*<0.00001 (z=15.7)
	x1/10 (415 nM)	25.66	16.77	N=228	*p*<0.00001 (z=-14.8)
	x1/100 (41.5 nM)	15.36	11.39	N=317	*p*<0.00001 (z=10.8)
	x1/200 (20.75 nM)	15.9	14.34	N=308	*p*<0.00001 (z=10.3)
	x1/400 (10.38 nM)	12.18	12.05	N=202	*p*<0.00001 (z=-6.94)
	x1/600 (6.92 nM)	11.82	12.03	N=238	*p*<0.00001 (z=5.33)
	x1/800 (5.19 nM)	8.83	9.09	N=201	*p*=0.35758 (z=-0.92)
	x1/1000 (4.15 nM)	8.69	9.78	N=206	*p*=0.88076 (z=0.15)
	x1/10000 (415 pM)	8.28	8.14	N=223	*p*=0.1902 (z=-1.31)

	Item on the x-axis	Cell length				Cell width		
		Average (μm)	SD (μm)	Cell num.	Mann-Whitney U test (Significance *p*<0.05) Against the 0h sample	Average (μm)	SD (μm)	Mann-Whitney U test (Significance *p*<0.05) Against the 0h sample
Fig. 3B and C	Sabouraud-0h	10.34	11.19	N=278		3.02	0.64	
	Sabouraud-8h	8.24	8.57	N=239	*p*=0.12852 (z=1.52)	3.48	0.76	*p*<0.00001 (z=-6.95)
	Sabouraud-24h	6.54	3.63	N=225	*p*<0.00001 (z=4.22)	3.31	0.47	*p*<0.00001 (z=-6.24)
	Sabouraud-48h	11.45	15.62	N=250	*p*=0.82588 (z=-0.22)	3.53	0.78	*p*<0.00001 (z=-8.05)
	Sabouraud-72h	23.63	37.7	N=279	*p*=0.0083 (z=2.64)	3.25	0.71	*p*<0.00001 (z=3.54)
	Sabouraud-96h	40.27	48.94	N=260	*p*<0.00001 (z=-8.88)	3.15	0.66	*p*<0.00001 (z=-2.09)
	Sabouraud-120h	61.48	68.47	N=213	*p*<0.00001 (z=-11.5)	3.38	0.81	*p*<0.00001 (z=-5.06)
	Sabouraud-144h	68.16	71.66	N=263	*p*<0.00001 (z=-14.4)	3.81	0.97	*p*<0.00001 (z=-10.12)
	Item on the x-axis	Average (μm)	SD (μm)	Cell num.	Mann-Whitney U test (Significance *p*<0.05) Against the 0h sample	Average (μm)	SD (μm)	Mann-Whitney U test (Significance *p*<0.05) Against the 0h sample
	Sabouraud+Mg-0h	10.34	11.19	N=278		3.02	0.64	

	Sabouraud+Mg-8h	13.06	6.38	N=232	$p<0.00001$ (z=-8.92)	3.57	0.77	$p<0.00001$ (z=-8.03)
	Sabouraud+Mg-24h	48.65	41.86	N=247	$p<0.00001$ (z=-16.5)	2.77	0.62	$p<0.00001$ (z=5.29)
	Sabouraud+Mg-48h	88.22	81.21	N=224	$p<0.00001$ (z=-15.0)	2.31	0.51	$p<0.00001$ (z=12.44)
	Sabouraud+Mg-72h	68.78	91.57	N=256	$p<0.00001$ (z=-9.49)	2.56	0.63	$p<0.00001$ (z=8.77)
	Sabouraud+Mg-96h	66.68	94.65	N=270	$p<0.00001$ (z=-11.0)	2.62	0.66	$p=0.02382$ (z=2.26)
	Sabouraud+Mg-120h	64.12	93.54	N=264	$p<0.00001$ (z=-10.8)	2.65	0.62	$p<0.00001$ (z=7.41)
	Sabouraud+Mg-144h	60.16	98.18	N=314	$p<0.00001$ (z=-12.1)	2.63	0.64	$p<0.00001$ (z=-8.2)

	Item on the x-axis	Average (μm)	SD (μm)	Cell num.	Mann-Whitney U test (Significance $p<0.05$)
Fig. 4B	18hr	9.49	8.67	N=251	
	38hr	49.07	48.38	N=257	$p<0.00001$ (z=13.93)

Cell length classification

	Item on the x-axis	0-10 μm	10-20 μm	20-30 μm	30-40 μm	40-50 μm	50-60 μm	60-70 μm	70-80 μm	80-90 μm	90-100 μm	>100 μm
Fig. 4B	18hr	192	39	10	4	2	4	0	0	0	0	0
	38hr	49	35	37	25	20	16	14	11	7	11	32

		Vacuolar area in Sabouraud medium (μm^2)			
	Item on the x-axis (cellular area)	Vacuolar size	Average (μm^2)	SD (μm^2)	Cell num. (cells including the indicated vacuole)
Fig. 5C	0-50 μm^2	more than 10 μm^2	0	0	N=35 (n=0)
		less than 10 μm^2	5.52	3.88	N=35 (n=29)
	50-100 μm^2	more than 10 μm^2	0.68	2.72	N=17 (n=1)
		less than 10 μm^2	11.63	5.59	N=17 (n=17)
	100-150 μm^2	more than 10 μm^2	1.44	3.81	N=16 (n=2)
		less than 10 μm^2	17.81	7.26	N=16 (n=16)
	150-200 μm^2	more than 10 μm^2	4.74	6.91	N=12 (n=4)
		less than 10 μm^2	23.01	8.3	N=12 (n=12)
	200-250 μm^2	more than 10 μm^2	2.56	5.71	N=6 (n=1)
		less than 10 μm^2	22.94	6.73	N=6 (n=6)
	250- μm^2	more than 10 μm^2	ND	ND	ND
		less than 10 μm^2	ND	ND	ND

		Vacuolar area in Sabouraud+Mg medium (μm^2)			
	Item on the x-axis (cellular area)	Size	Average (μm^2)	SD (μm^2)	Cell num. (cells including the indicated vacuole)
Fig. 5C	0-50 μm^2	more than 10 μm^2	0	0	N=19 (n=0)
		less than 10 μm^2	5.69	3.8	N=19 (n=19)
	50-100 μm^2	more than 10 μm^2	6.45	6.04	N=13 (n=7)
		less than 10 μm^2	11.95	7.62	N=13 (n=13)
	100-150 μm^2	more than 10 μm^2	11.03	9.09	N=14 (n=9)
		less than 10 μm^2	21.76	9.94	N=14 (n=14)
	150-200 μm^2	more than 10 μm^2	29.63	21.62	N=14 (n=12)
		less than 10 μm^2	24.26	13.08	N=14 (n=14)
	200-250 μm^2	more than 10 μm^2	39.12	19.44	N=10 (n=9)
		less than 10 μm^2	35.65	9.93	N=10 (n=10)
	250- μm^2	more than 10 μm^2	143.94	93.59	N=16 (n=16)
		less than 10 μm^2	37.09	13.39	N=16 (n=16)

	Item on the x-axis	Average (μm)	SD (μm)	Cell num.	Mann-Whitney U test (Significance $p<0.05$)	
					Against the LA10 sample	Against the DMSO sample
Fig.6A	Sabouraud+Mg+DMSO	56.32	51.51	N=247	$p<0.00001$ (z=-16.51)	
	Sabouraud+Mg+BZ100	56.17	50.1	N=251	$p<0.00001$ (z=-16.55)	$p=0.98404$ (z=-0.022)
	Sabouraud+Mg+LA10	11.96	8.69	N=360		$p<0.00001$ (z=-16.51)
	Item on the x-axis	Average (μm)	SD (μm)	Cell num.	Mann-Whitney U test (Significance $p<0.05$)	
					Against the LA10 sample	Against the DMSO sample
	YPD+DMSO	22.39	10.26	N=277	$p<0.00001$ (z=-10.36)	
	YPD+BZ100	22.59	20.28	N=305	$p<0.00001$ (z=-8.93)	$p=0.00804$ (z=-2.65)
	YPD+LA10	15.87	13.22	N=306		$p<0.00001$ (z=-10.36)

Cell length classification

	Item on the x-axis	0-10 μm	10-20 μm	20-30 μm	30-40 μm	40-50 μm	50-60 μm	60-70 μm	70-80 μm	80-90 μm	90-100 μm	>100 μm
Fig. 6A	Sabouraud+Mg+DMSO	20	39	27	36	27	18	14	13	10	8	35
	Sabouraud+Mg+BZ100	17	51	33	23	18	16	15	18	13	8	39
	Sabouraud+Mg+LA10	201	120	24	10	1	1	1	2	0	0	0
	Morphology index											
	Item on the x-axis	0-10 μm	10-20 μm	20-30 μm	30-40 μm	40-50 μm	50-60 μm	60-70 μm	70-80 μm	80-90 μm	90-100 μm	>100 μm
	YPD+DMSO	18	121	86	35	9	7	1	0	0	0	0
	YPD+BZ100	22	160	78	26	6	2	5	2	0	1	3
	YPD+LA10	102	141	43	6	6	1	0	4	1	1	1

		YPD			
	Item on the x-axis (cell length)	Phenotype A	Phenotype B	Phenotype C	Classification of mitochondrial distribution
Fig. 7C	0-10 μm	22	1	9	Phenotype A: Distribution throughout the cell cytoplasm and adjacent to the cell walls
	10-20 μm	133	0	1	Phenotype B: Fragmented distribution
	20-30 μm	92	0	0	Phenotype C: Reduced distribution
	30-40 μm	20	0	0	
	40-50 μm	5	0	0	
	50- μm	2	0	0	

		Sabouraud			
	Item on the x-axis (cell length)	Phenotype A	Phenotype B	Phenotype C	Classification of mitochondrial distribution
Fig. 7C	0-10 μm	32	88	62	Phenotype A: Distribution throughout the cell cytoplasm and adjacent to the cell walls
	10-20 μm	23	21	9	Phenotype B: Fragmented distribution
	20-30 μm	8	7	4	Phenotype C: Reduced distribution

	30-40 μm	1	2	1
	40-50 μm	1	0	1
	50- μm	0	0	0

		Sabouraud+Mg			
	Item on the x-axis (cell length)	Phenotype A	Phenotype B	Phenotype C	Classification of mitochondrial distribution
Fig. 7C	0-10 μm	30	17	33	Phenotype A: Distribution throughout the cell cytoplasm and adjacent to the cell walls
	10-20 μm	40	1	8	Phenotype B: Fragmented distribution
	20-30 μm	33	1	0	Phenotype C: Reduced distribution
	30-40 μm	24	0	0	
	40-50 μm	21	0	0	
	50- μm	28	0	0	

		Cell		Vacuolar area (μm^2)	
		Cell number	Cellular area	Average	SD
Fig. S4B	SYTOX-positive cells	N=89	0-50 μm^2	0.98	1.23
		N=13	50- μm^2	5.35	4.35
		N=102	All	1.54	2.42
	SYTOX-negative cells	N=93	0-50 μm^2	4.05	2.83
		N=7	50- μm^2	14.13	13.74
		N=100	All	4.76	5.22

Values of average cell length, standard deviation, cell number, and *p*-value, that were used in Fig. 2A, 2B, 2C, 2D, 3B, 4B, 5C, 6A, and 7C were described. Actual values in the cell length classification were described. LA10 is 10 μg/ml latrunculin A. Statistical differences between samples were calculated using Mann–Whitney U Test. The *p*-values are significant at $p < 0.05$.
